# Bedside Laparoscopy in the Critically Ill: A Review of the Literature

**DOI:** 10.3390/jcm13061530

**Published:** 2024-03-07

**Authors:** Alessandro Palladino, Carlo Vallicelli, Daniele Perrina, Girolamo Convertini, Federico Coccolini, Luca Ansaloni, Massimo Sartelli, Fausto Catena

**Affiliations:** 1 Department of General, Emergency, and Trauma Surgery, M. Bufalini Hospital, 47521 Cesena, Italy; alessandro.palladino@auslromagna.it (A.P.); carlo.vallicelli@auslromagna.it (C.V.); perrinadaniele@gmail.com (D.P.); girolamo.convertini@gmail.com (G.C.); 2 Department of General, Emergency and Trauma Surgery, University Hospital of Pisa, 56124 Pisa, Italy; federico.coccolini@gmail.com; 3 Department of General, Emergency and Trauma Surgery, IRCCS Policlinico San Matteo Foundation, University of Pavia, 27100 Pavia, Italy; aiace63@gmail.com; 4 Department of Surgery, Macerata Hospital, 62100 Macerata, Italy; m.sartelli@virgilio.it

**Keywords:** bedside laparoscopy, minimally invasive surgery, critically ill patients

## Abstract

Critically ill patients treated in the intensive care unit (ICU) can present with many abdominal conditions that need a prompt diagnosis and timely treatment because of their general frailty. Clinical evaluation and diagnostic tools like ultrasound or CT scans are not reliable or feasible in these patients. Bedside laparoscopy (BSL) is a minimally invasive procedure that allows surgeons to assess the abdominal cavity directly in the ICU, thus avoiding unnecessary exploratory laparotomy or incidents related to intra-hospital transfer. We conducted a review of the literature to summarize the state-of-the-art of BSL. The Medline, Cochrane Central Register of Controlled Trials (CENTRAL), and Scopus databases were utilized to identify all relevant publications. Indications, contraindications, technical aspects, and outcomes are discussed. The procedure is safe, feasible, and effective. When other diagnostic tools fail to diagnose or exclude an intra-abdominal condition in ICU patients, BSL should be preferred over exploratory laparotomy.

## 1. Introduction

Critically ill patients treated in the intensive care unit (ICU) can present with many abdominal conditions [1] that need a prompt diagnosis and timely treatment because of their general frailty. Clinical evaluation is not always reliable due to sedation or analgesia. In some cases, it can be misleading, causing a delayed diagnosis, which can be detrimental to this category of patient [2]. Furthermore, the accuracy of diagnostic tests such as ultrasound or CT scans is reduced. In particular, ultrasound benefits from patient compliance and is operator-dependent [3], while a CT scan requires intra-hospital transportation with its established rates of related complications and mortality [4,5]. These include patient-related or equipment-related adverse events such as hypotension, respiratory distress, dysrhythmias, central line disconnections, oxygen supply problems, or accidental extubation.

Given the physiological derangement of ICU patients, the impaired ability to perform a physical examination, the reduced accuracy of diagnostic tests, and the time-dependency imposed by these situations, the treatment of choice—when a diagnosis has not been established—has historically been an exploratory laparotomy. To date, there are no data regarding the rate of positive intra-abdominal findings in exploratory laparotomies in critically ill patients. Nonetheless, a negative laparotomy is a reality that surgeons encounter in their practice, and some reports on other types of patients [6,7,8,9] suggest that this rate and the associated comorbidities are far from negligible. In a retrospective analysis of 817 patients who underwent an exploratory laparotomy after abdominal gunshot wounds, Sosa et al. found a negative laparotomy to be associated with a 22% morbidity rate and an average length of stay of 5.1 days, while Weigelt et al., in a retrospective study enrolling 248 patients, reported a 14% postoperative morbidity rate with a mean hospital stay of 5 days [7,9].

Bedside laparoscopy (BSL) is a minimally invasive procedure that allows surgeons to assess the abdominal cavity directly in the ICU, thus avoiding unnecessary exploratory laparotomy or incidents related to intra-hospital transfer.

The present review summarizes the state of the art of BSL.

## 2. Materials and Methods

We searched through the Medline, Cochrane Central Register of Controlled Trials (CENTRAL), and Scopus databases using the following terms: “bedside laparoscopy”, “diagnostic laparoscopy”, “critically ill”, “ICU”, and “intensive care unit”. MeSH terms were included. We also conducted a hand-search of references of previous reviews on the same topic to include all relevant studies. We included all studies reporting on patients undergoing BSL only in the ICU; therefore, we excluded studies in which a diagnostic laparoscopy was carried out in the operating room, even though it was performed on an ICU patient.

## 3. Indications and Contraindications

The physiological consequences of pneumoperitoneum, especially in critically ill patients, allow only for brief and basic operations, as they may outweigh the benefits of BSL in the case of time-consuming or technically challenging procedures [10,11]. Therefore, BSL should be primarily used to recognize intra-abdominal pathology not identified by other diagnostic tools or to perform short and simple operations such as controlling minor bleeding or placing a drain. By following these recommendations, BLS can effectively lower the rate of unnecessary exploratory laparotomy while keeping the instrumentation requirements and the possible complications to a minimum.

The Society of American Gastrointestinal and Endoscopic Surgeons (SAGES) guidelines state that the indications for diagnostic laparoscopy in acute conditions in which a diagnosis has not been established are trauma, acute abdominal pain, and ICU patients [12]. In the first two categories, diagnostic laparoscopy is used only in hemodynamically stable patients [13]; hence, the procedure is generally performed in the operating room. Conversely, for critically ill patients, BSL can and should be conducted in the ICU for the following reasons:

1. Unexplained sepsis, systemic inflammatory response syndrome, or multiorgan failure;
2. Abdominal pain or tenderness associated with other signs of sepsis without an obvious indication for laparotomy;
3. Increased abdominal distention that is not a consequence of bowel obstruction;
4. Fever or leukocytosis in an obtunded or sedated patient not explained by another identifiable problem;
5. Metabolic acidosis not explained by another process.

While BSL represents an effective tool in the diagnostic process in the situations mentioned above, surgeons should bear in mind that it is still an invasive procedure with well-known cardiac and respiratory consequences in an already frail population. Thus, thorough patient selection is mandatory. When planning a BSL, the following contraindications should be considered:

1. Patients unable to tolerate pneumoperitoneum or who are so critical that they have no chance of survival even if a treatable intra-abdominal pathology is found;
2. Patients with an already established diagnosis requiring surgical intervention;
3. Patients with uncorrected coagulopathy or hypercapnia;
4. Patients with suspected abdominal compartment syndrome;
5. Patients who have undergone a laparotomy in the last 4–6 weeks;
6. Patients with intra-abdominal adhesions secondary to previous surgical interventions (relative).

## 4. Instrumentation and Setting

As mentioned above, the severe clinical conditions faced by most patients admitted to the ICU force BSL to be a concise and straightforward procedure. Therefore, the required instrumentation reflects this necessity. In the published series [14,15,16,17,18,19,20,21,22,23,24], surgeons utilized a basic laparoscopic set including a monitor, an insufflator, and a light source, all placed on a dedicated mobile tower stored in the ICU, a 30° scope, a light cord, a Hasson trocar (10–12 mm) and two 5 mm trocars, manipulating instruments, and standard sterile dressing and drapes. The procedure was conducted in an isolated single bedroom in the ICU ward. All the staff in the room—including an anesthesiologist, a surgeon and an assistant, one nurse from the operating room, and one ICU nurse—wore protective clothing, surgical masks and caps, and gloves. Standard operating room protocols were meticulously followed to ensure sterility.

## 5. Anesthesiological Technique

Eight of the included studies reported details on the anesthesiological technique [14,15,16,17,18,21,22,23,24]. An anesthesiologist directed the general anesthesia induction, mechanical ventilation, and hemodynamic support of the patients during the procedure in all cases except for Karasakalides et al. and Kelly et al.’s work [18,24], in which the ICU nurse was responsible for patient monitoring. The vast majority of patients were already intubated. Patients not requiring mechanical ventilation were generally intubated just before the procedure. Only Pecoraro et al. did not insert an endotracheal tube in patients not artificially ventilated before BSL [22]. General anesthesia was performed in a total intravenous fashion via a bolus of propofol, midazolam, ketamine, and remifentanil or fentanyl. A neuromuscular blockade was achieved with either atracurium or cisatracurium. When needed, hemodynamic support was ensured with noradrenaline or dobutamine infusion.

## 6. Surgical Technique

Patients were placed in their ICU bed in a supine position; Trendelemburg and anti-Trendelemburg inclinations were used to explore the entire abdominal cavity. A standard surgical field with abdominal prepping and surgical dressing and drapes was created following operating room protocols. The pneumoperitoneum was achieved using an open Hasson technique with a 10–12 mm trocar placed in the paraumbilical region. However, the suspected presence of intra-abdominal adhesions should dictate the placement of the first trocar, aiming for a so-called “virgin” site to minimize the risk of a hollow viscus injury. Only Jaramillo et al. used a Veress needle to insufflate the abdominal cavity [16].

When reported, intra-abdominal pressure was generally maintained between 8 and 10 mmHg to minimize the physiological consequences of pneumoperitoneum, especially with concerns about hemodynamic instability in an already compromised population. Peris et al. reported a pressure of 8 to 15 mmHg [15], while Walsh et al. set the intra-abdominal pressure at 15 mmHg [17]. Once the pneumoperitoneum was established, two additional 5 mm trocars were generally placed. These two additional working ports were often inserted “as needed” without a standardized technique. Alemanno et al. reported the insertion of two 5 mm trocars in the left part of the abdomen to explore the right quadrants and two 5 mm trocars on the right part of the abdomen to explore the left quadrants [14], while Hackert et al. and Bergamini et al. used two 5 mm trocars in the left and right lower quadrants [19,21]. The mean procedure time was tracked in seven of the included studies; only Hackert et al., Bergamini et al., Ceribelli et al., and Kelly et al. did not report it [19,21,23,24]. The mean operative time ranged from 19 to 40 min. Karasakalides et al. also performed a CT scan prior to BSL, finding BSL to be a less time-consuming procedure requiring, on average, 21 min, while a CT scan took 38 min.

## 7. Patients Characteristics and Outcomes

In the included studies [14,15,16,17,18,19,20,21,22,23,24,25], 399 patients underwent BSL. The most frequent ICU admission diagnosis was by far post-cardiac surgery monitoring, with 202 cases (50.6% of patients), followed by sepsis (68 cases), trauma (41 cases), vascular surgery (16 cases), respiratory failure (12 cases), cardiogenic shock (12 cases), and some other less frequent etiologies. The patient characteristics in the included studies are summarized in Table 1. These results are partly biased because of the studies by Hackert et al. [19] and Bergamini et al. [21], which enrolled only patients admitted to the ICU after a cardiac surgery procedure. Nonetheless, gastrointestinal complications after cardiac surgery are rare but devastating occurrences often burdened by serious clinical sequelae and high mortality rates, with incidence rates ranging from <1% to 4.1% and mortality rates ranging between 13.9% and 63% [26,27]. In a retrospective analysis of 4819 patients undergoing cardiac surgery, Filsoufi et al. found age, myocardial infarction, congestive heart failure, hemodynamic instability, cardiopulmonary bypass time > 120 min, peripheral vascular disease, renal and hepatic failure as independent predictors of gastrointestinal complications [28].

An intra-abdominal pathology was found in 210 of these patients, with positive finding rates ranging from 30.2% to 88.2%. By far the two most frequent diagnoses after BSL were bowel ischemia/hypoperfusion, discovered in 93 cases with rates ranging from 11.2% to 66.7%, and acute cholecystitis, found to be the underlying cause of clinical worsening in 64 patients, with rates ranging from 0% to 67.4%. Together, they accounted for 74.7% of all positive findings and were present in 39.3% of all BSLs performed. Other positive intra-abdominal explorations were due to abnormal colonic distension (four cases), post-traumatic occult injuries (three cases), a perforated peptic ulcer (three cases), acute pancreatitis (two cases), and liver ischemia (two cases). Interestingly, peritonitis (either purulent or fibrinous), without an identifiable cause, was the only abnormal finding in BSL in 11 patients. BSL also showed high sensitivity rates, with percentages equal to or above 90% in the three studies that confirmed BSL findings with either laparotomy or autopsy [16,19,24]. It is to be noted that although these sensitivity rates are comparable to those of diagnostic laparoscopy and, therefore, could be considered representative of the actual rates, the total sample size of the studies by Jaramillo et al., Hackert et al., and Kelly et al. is 32 patients. Further studies, including a more significant number of patients, are needed to confirm these results.

The most favorable feature of BSL is its low invasiveness, allowing the diagnosis or exclusion of intra-abdominal pathologies while avoiding exploratory laparotomy in patients in whom other diagnostic tools are unfeasible or inconclusive. Literature data indicate that an unnecessary exploratory laparotomy can be avoided in 30.6–91.6% of cases. Excluding data from Jaramillo et al. and Hackert et al., which did not report the percentage of patients not necessitating a subsequent laparotomy, 226 out of 309 patients did not undergo an exploratory laparotomy.

After a positive BSL, the treatment was mainly operative—either radiologic, laparoscopic, or laparotomic—with rates ranging from 20.0% to 100.0%. Overall, 0.0–21.4% of patients were treated conservatively. There was also a considerable rate of palliative support, which is to be expected in critically ill patients admitted to the ICU ward.

Mortality rates were also significantly high in patients with positive findings in BSL, ranging from 33.3% to 80.0%. No deaths occurred during or as a consequence of BSL. Therefore, these rates are not related to BSL itself but rather to the frail conditions of these patients.

The safety profile of BSL in the included studies was noticeable. Most of the studies reported no complications, while in the other studies, the most frequent post-procedural complication was superficial wound infection. No major complication (Clavien-Dindo > II [25]) was observed.

## 8. Limitations

In all the included studies, the authors pointed out that the main limitation of BSL is the exploration of the retroperitoneal compartment. Although this statement is undoubtedly true, the present review has demonstrated that in 74.7% of patients, the unknown intra-abdominal pathology is either bowel ischemia/hypoperfusion or acute cholecystitis. Furthermore, out of the 210 positive BSL findings, only in 2 patients was a retroperitoneal pathological condition (acute pancreatitis) responsible for the patient’s clinical deterioration.

It is also to be noted that in 11 cases, BSL was able to find peritonitis, either fibrinous or purulent, without an apparent underlying cause. It can be inferred that, in these 11 patients, the peritonitis had an unidentified origin and that BSL was inconclusive or nondiagnostic rather than considering them as cases of “spontaneous” peritonitis.

## 9. BSL vs. CT Scan

A suspected intra-abdominal pathology in an already compromised population, such as ICU patients, should not be underestimated because, if not treated swiftly, it could lead to a rapid and severe worsening of the clinical conditions and, in some cases, to death. It is generally investigated with a radiological examination, such as a CT scan of the abdomen, although this technique has different sensitivity and specificity rates for each possible cause. For example, Menke, in his meta-analysis, studied the accuracy of CT in detecting acute mesenteric ischemia for all causes, showing a pooled sensitivity of 93% and a pooled specificity of 95% [29]. When considering the use of abdominal CT scans in patients undergoing cardiac surgery, which represents the most common admission diagnosis in patients undergoing BSL, it should be remembered that the most frequent cause of acute mesenteric ischemia is non-occlusive mesenteric ischemia (NOMI) given the impaired cardiac output particular to this group of patients, with rates ranging from 48% to 83% [30,31]. Despite the great accuracy demonstrated by CT scans in detecting acute mesenteric ischemia for all causes, when evaluating only NOMI, CT scans are not as efficient and could potentially underestimate an ongoing ischemic injury [32,33].

Although CT scans have traditionally been thought to be less sensitive than ultrasound for diagnosing acute cholecystitis [34], recent data have found that CT scans are more reliable in detecting acute cholecystitis [35]. However, in critically ill patients, the most common cause of acute cholecystitis is acalculous cholecystitis, a clinical entity for which the diagnostic accuracy of CT scans has yet to be studied.

Given that the two most common positive findings in BSL are acute mesenteric ischemia and acute cholecystitis and that the most common underlying causes of these two clinical entities in critically ill patients are NOMI and acute acalculous cholecystitis, it is safe to assume that in this subgroup of patients, CT scans have lower efficiency. In a 2015 study by Just et al. analyzing the role of CT scans in identifying a potential infectious source in critically ill patients, CT scans detected an infectious source in 52.8% of cases, resulting in a change in treatment in 85.5% of cases. Conversely, the treatment was changed in 16.2% of cases in patients without identification of an infectious source. In their conclusions, the authors called for a more careful and standardized use of CT scans in ICU patients [36]. In their clinical practice, when evaluating intra-abdominal pathologies in critically ill patients, surgeons should bear in mind the lower accuracy of CT scans and consider BSL in cases of an inconclusive clinical examination and radiology.

## 10. Discussion

Since its introduction, laparoscopy has greatly enhanced surgical patient outcomes, determining a significant reduction in postoperative morbidity, pain, and length of stay, granting a less uncomfortable and more speedy recovery while, at the same time, being not inferior to the laparotomic approach in all other surgical aspects. On the contrary, when treating critically ill patients, the use of laparoscopy is often, at least in the authors’ opinion, unfairly underrated. To this date, when addressing a suspected intra-abdominal condition, surgeons and anesthesiologists are skeptical regarding the potential benefits of laparoscopy and instead prefer a laparotomic approach, fearing the effect of the well-known cardiac and respiratory consequences of pneumoperitoneum in a frail and already compromised population.

In the present review, all aspects revolving around BSL were covered. The procedure can be conducted safely in a brief but thorough manner directly in the ICU, avoiding a superfluous transportation in those with negative findings in BSL or effectively diagnosing an intra-abdominal pathology allowing transportation and operational treatment (either radiologic, laparoscopic, or laparotomic) only for patients who actually require them. In the included series, an unnecessary laparotomy was avoided in 226 out of 309 patients (73.1%), either due to negative findings, due to the diagnosis of an untreatable condition, or due the diagnosis of a treatable condition not necessitating a laparotomy. Although not explicitly reported, it is possible that laparotomy was considered the only treatment option after a positive finding in BSL in some of the included works. To our knowledge, the optimal approach after a positive finding in BSL is yet to be studied, and, therefore, no standardized algorithm exists. The present analysis shows that laparoscopy can be safely utilized in critically ill patients and, therefore, employed not only as a diagnostic tool but also as a treatment option, confining the role of laparotomy only to those patients who cannot withstand a more prolonged laparoscopic procedure. The latter needs to be confirmed by further studies directly addressing the outcomes of laparoscopy compared to laparotomy in these patients.

As already stated above, the safety profile of BSL is remarkable, showing very low procedure-related morbidity rates, with the most common complication being superficial wound infection. Also, mortality rates, although undoubtedly high, are not to be ascribed to BSL but instead to the poor general conditions particular to ICU patients. The low procedure-related morbidity and mortality rates should further encourage surgeons to use BSL.

The present review has some limitations. The included studies are all retrospective series with generally a low number of patients. Furthermore, a control group is, in most cases, absent. This precluded us from carrying out a systematic review on the topic which would have represented stronger evidence in favor of BSL. Nonetheless, our work sheds light on the benefits of the use of BSL in critically ill patients, when other diagnostic tools have failed to obtain a diagnosis. These findings need to be supported by studies with a larger sample size and a control group.

## 11. Conclusions

The present review summarizes the state of the art of BSL. The procedure is safe, feasible, and effective. When other diagnostic tools fail to diagnose or exclude an intra-abdominal condition in ICU patients, BSL should be preferred over exploratory laparotomy.

## Figures and Tables

**Table 1:** Summary of the included studies.

Study	Year	Patients	Admission Diagnosis (n)	Positive Findings (%)	Diagnosis (n)	Unnecessary Laparotomy Avoided (%)	Treatment (n)	Mortality (BSL +)
Alemanno [14]	2019	129	Cardiac surgery (90) Sepsis (25) Trauma (14)	69 (53.5)	Bowel ischemia (40) Bowel hypoperfusion (6) Cholecystitis (13) Bowel perforation (2) Purulent peritonitis (4) Other (4)	55.0	Operative (58) Conservative (6) Palliative (5)	40.6
Peris [15]	2009	32	Cardiac surgery (6) Sepsis (12) Trauma (14)	15 (46.8)	Cholecystitis (7) Bowel ischemia (1) Bowel perforation (2) Bowel hypoperfusion (2) Purulent peritonitis (3)	75.0	Operative (8) PGBD (5) Conservative (2)	33.3
Jaramillo [16]	2006	13	Sepsis (7) MOF (1) Acute myocardial infarction (2) Intestinal obstruction (1) Bowel ischemia (2)	9 (69.2)	Bowel ischemia (6) Bowel perforation (1) Cholecystitis (2)	/	Operative (2) Palliative (7)	77.8
Walsh [17]	1998	12	Cardiac surgery (1) Cardiac (not surgery) (3) Sepsis (5) Respiratory failure (2) Aortic occlusion (1)	5 (41.6)	Bowel ischemia (2) Sigmoid diverticulitis (1) Terminal ileitis (1) Non-purulent peritonitis (1)	91.6	Operative (1) Palliative (4)	80.0
Karasakalides [18]	2009	35	Trauma (5) Respiratory failure (9) Cardiogenic shock (8) Sepsis (11) Cerebral hemorrage (2)	15 (42.8)	Bowel ischemia (6) Cholecystitis (5) Biliary peritonitis (1) Purulent peritonitis (2) Mesenteric hemorrage (1)	82.8	Operative (11) Palliative (4)	60.0
Hackert [19]	2003	17	Cardiac surgery (17)	15 (88.2)	Bowel ischemia (6) Bowel distension (4) Acute cholecystitis (3) Fibrinous peritonitis (1) Acute pancreatitis (1)	/	Operative (14) Conservative (1)	53.3
Sajid [20]	2018	28	/	14 (50.0)	Bowel ischemia (7) Cholecystitis (2) Liver ischemia (2) Acute pancreatitis (1) Gangrenous uterus (1) Gastric volvulus (1)	82.1	Opertive (8) Conservative (3) Palliative (3)	/
Bergamini [21]	2022	43	Cardiac surgery (43)	13 (30.2)	Bowel ischemia (13)	69.7	/	46.1
Pecoraro [22]	2001	11	Sepsis (4) Intestinal perforation (3) Bowel ischemia (1) Thoracic surgery (1) Diabetic ketoacidosis (1) Jaundice (1)	6 (54.5)	Duodenal perforation (1) LUQ abscess (1) Large pelvic mass (1) Cirrhosis (1) Bowel ischemia (1) Colo-vesical fistula (1)	63.6	Operative (4) Percutaneous drainage (1) Conservative (1)	50.0
Ceribelli [23]	2012	62	Cardiac surgery (38) Vascular surgery (13) Trauma (6) Sepsis (3) Neurosurgery (2)	43 (69.3)	Cholecystitis (29) Bowel ischemia (5) Diaphragmatic rupture (3) Perforated peptic ulcer (2) Minor hepatic/splenic injury (2) Aorto-aortic anastomosis leak (2)	30.6	Operative (43)	48.8
Kelly [24]	2000	17	Cardiac surgery (7) Vascular surgery (3) Trauma (2) Neurosurgery (1) Urologic surgery (1) ESRD (1) Cardiac (not surgery) (1) Respiratory failure (1)	6 (35.3)	Bowel ischemia (4) Cholecystitis (2)	70.6	Operative (6)	50.0

BSL: bedside laparoscopy; PGBD: percutaneous gallbladder drainage; ESRD: end-stage renal disease.

## Data Availability

The raw data supporting the conclusions of this article will be made available by the authors on request.
